# Huangyusang decoction for Type 2 diabetes: A protocol for systematic review and meta analysis

**DOI:** 10.1097/MD.0000000000024576

**Published:** 2021-02-26

**Authors:** Peiyu He, Junyin Zhang, Tianyu Gao, Yingxiang Wang, Teng Peng

**Affiliations:** School of Pharmacy, Key Laboratory of Standardization of Chinese Medicinal Materials, Ministry of Education, Chengdu University of Traditional Chinese Medicine, Chengdu, Sichuan, China.

**Keywords:** β-cell dysfunction, Huangyusang decoction, protocol, systematic review, type 2 diabetes

## Abstract

**Background:**

Diabetes is a chronic metabolic disease characterized by elevated blood glucose levels due to insulin resistance and β-cell dysfunction. In China, Huangyusang decoction (HYS) has been widely used to treat Type 2 diabetes. However, there is no systematic review found. In order to evaluate the efficacy and safety of HYS in the treatment of Type 2 diabetes, we need to conduct a meta-analysis and systematic evaluation.

**Methods:**

We will enroll the randomized controlled trials (RCTs) evaluating the effectiveness and safety of HYS in the treatment of Type 2 diabetes. Data come mainly from 4 Chinese databases (CNKI, Wanfang, CBM, and VIP Database) and 4 English databases (PubMed, Embase, Cochrane Library, and Web of science). The enrollment of RCTs is from the starting date of database establishment till January 30, 2021. Fasting blood glucose is considered as the main indicator of the dyslipidemia, while the body mass index, glycated hemoglobin, fasting insulin, triglycerides, and cholesterol are regarded as the secondary indicators. There are safety indicators including liver enzyme and kidney function. The work such as selection of literature, data collection, quality evaluation of included literature, and assessment of publication bias will be conducted by 2 independent researchers. Meta-analysis will be performed by RevMan 5.0 software.

**Results:**

This study will provide high-quality evidence for the effectiveness and safety of HYS in the treatment of type 2 diabetes.

**Conclusion:**

The results of the study will help us determine whether HYS can effectively treat type 2 diabetes.

**Ethics and dissemination:**

This study does not require ethical approval. We will disseminate our findings by publishing results in a peer-reviewed journal.

**OSF registration number:**

DOI 10.17605/OSF.IO/AXBRV.

## 1 Introduction

Type 2 diabetes mellitus (T2DM), which is characterized by insulin resistance, β-cell failure and low-grade inflammation, is a major health risk worldwide.^[1]^ Data have shown that the world population is facing a surge in T2DM and prediabetes due to rapid changes in lifestyle, including a growing availability of food high in calories and a decline in physical activity.^[2]^ Approximately 5% of the population worldwide has been diagnosed as suffering from T2DM, and this level is projected to increase to 9.9% by 2030.^[3]^ For patients whose diabetic duration is less than 5 years, DR morbidity is approximately 38% to 39%, and for those whose duration is 5 to 10 years, the morbidity is approximately 50% to 56.7%, whereas for patients whose diabetic duration is more than 10 years, the morbidity is approximately 69% to 90%.^[4]^ Insulin, Sulfonylureas, and Biguanides are traditional hypoglycemic drugs. The key to the treatment of diabetes is to reduce blood glucose and fasting blood glucose. The use of conventional drugs also produces side effects, including hypoglycemia, gastrointestinal discomfort, nausea, liver failure and heart failure, and diarrhea.^[5–8]^ These drugs are effective in lowering the glucose level of blood, but they also have some side effects and body dependence can develop.^[9]^ In this case, the hypoglycemic treatment with alternative drugs has attracted more and more attention. Many studies have shown that the HYS prescription represented by *Polygonatum sibiricum, Rhizoma Polygonati Odorati*, and *Folium Mori* has a commendable hypoglycemic effect.^[10–12]^ However, we have found no systematic study on the efficacy and safety of HYS lipid-lowering therapy. So, we will systematically evaluate the efficacy and safety of HYS in treating type 2 diabetes by a meta-analysis method, which provide strong evidence-based medicine support for its clinical applications.

## 2 Methods

### 2.1 Protocol and registration

The protocol has been registered on the Open Science Framework (OSF) platform (https://osf.io/axbrv/), registration number: DOI 10.17605/OSF.IO/AXBRV. This protocol was drafted and reported in accordance with the Preferred Reporting Items for Systematic Reviews and Meta-Analyses Protocols (PRISMA-P) guidelines.^[13]^ The final report will comply with the recommendations of the PRISMA Extension Statement for Reporting of Systematic Reviews Incorporating Meta-analyses of Healthcare Interventions.^[14]^

### 2.2 Ethics

We will not need individual data of each patient in the research, as this is a systematic review. Therefore, institutional review board approval and ethics committee is not needed. Our purpose is to publish the results in a peer-reviewed journal. The final results of the review will provide information about the safety and efficacy of HYS and its modified forms in the treatment of type 2 diabetes to help clinicians make decisions on clinical practice.

### 2.3 Eligibility criteria

The participant (P), intervention (I), comparator (C), outcome (O), and study design (S) are the 5 main factors determining the inclusion and exclusion criteria of this research.

#### 2.3.1 Type of study design

We will exclude quasi-randomized controlled trial (RCT), non-RCT, observation group combination other drugs, animal experiments, control groups not match, and full texts not available by reading the title, abstract, and related quotations information screening. Then, we can eliminate incomplete experimental data and experimental design schemes not rigorous, no clear diagnostic criteria by reading and understanding the full paper, and finally RCTs were included this research.

#### 2.3.2 Type of participant

Adult participants (older than 18 years) with type 2 diabetes and no other illness will be enrolled. The diagnosis of type 2 diabetes can be established if the patient’ FBG remain high 2 to 4 weeks later after his initial visit.^[15,16]^ No gender, race, nationality, and comorbidity are limited.

#### 2.3.3 Type of interventions

The patients in the treatment groups will be given HYS or modified HYS as a monotherapy or in combination with conventional therapy. HYS consisting of 3 herbs: *Polygonatum sibiricum, Rhizoma Polygonati Odorati*, Folium Mori. According to “Jun-Chen-Zuo-Shi” principle of Chinese herbal formula, *Polygonatum sibiricum* is “Jun” herb, and *Rhizoma Polygonati Odorati* and *Folium Mori* are “Chen” herb, both of which are the core of HYS. Therefore, modified HYS should include *Polygonatum sibiricum, Rhizoma Polygonati Odorati, Folium Mori* basically. The number of modified herbs will not exceed 3 (n ≤ 3). Patients of control group will be treated with conventional therapy with placebo, stains, and Biguanides. In addition, the 2 groups have not taken any drugs that possibly interfered with the outcome indicators. The follow-up time was ≥4 weeks.

#### 2.3.4 Type of outcomes

##### 2.3.4.1 Primary outcomes

According to the “Clinical Research Guidelines for New Chinese Medicines” and the new AACE/ACE guidelines, FBG is determined as the main indicator. If FBG decreases by ≥20%, it is considered to have a significant effect^[15,17]^; if FBG decreases by 10% to 20%, it is considered to be effective; invalid, if the level of FBG decline does not meet the above criteria.

##### 2.3.4.2 Secondary outcomes

According to the “Clinical Research Guidelines for New Chinese Medicines,”^[15]^ the secondary indicators include BMI, TG, TC, FINS.

##### 2.3.4.3 Safety outcomes

Safety indicators consist of liver enzyme, kidney function.

### 2.4 Literature retrieval strategy

Computer search of PubMed, the Cochrane Library, EMbase, China National Knowledge Infrastructure (CNKI), China Biomedicine (CBM), Chinese Scientific Journals Database (VIP), Wanfang Database for published information about RCTs of HYS for the treatment of type 2 diabetes. The time limit for literature search is from the establishment of each database to January 30, 2021. The language is limited to English and Chinese. In addition, the World Health Organization International Clinical Trials Registration Platform and Clinical Trials website (Clinical Trials.gov) will be searched for ongoing trials related to the disease and RCTs in China. The search method uses a combination of free words and medical subject terms, including “ type 2 diabetes,” “ pathoglycemia,” “spleen and stomach,” “Chinese medicine,” “huangyusang decoction,” etc. Chinese database search will use the following terms: “erxingtangniaobing,” “jiangtang,” “xuetangyichang,” “piwei,” “zhongyao,” “huangyusangtang,” etc. Taking PubMed as an example, the initial search strategy is summarized in Table 1, which will be adjusted according to the specific database.

**Table 1:** Search strategy of the PubMed.

Number	Search terms
#1	type 2 diabetes [Mesh]
#2	type 2 diabetes [Title/Abstract] OR type 2 diabetes [Title/Abstract] OR type 2 diabetes [Title/Abstract] OR pathoglycemia [Title/Abstract] OR pathoglycemia [Title/Abstract] OR pathoglycemia [Title/Abstract] OR pathoglycemia [Title/Abstract]
#3	#1 OR #2
#4	Huangyusang[Title/Abstract]
#5	Decoction[Title/Abstract]
#6	#4 AND #5
#7	randomized controlled trial[Publication Type]
#8	controlled clinical trial[Publication Type]
#9	randomized[Title/Abstract]
#10	randomly[Title/Abstract]
#11	#10 OR #11 OR #12 OR #13
#12	#3 AND #6 AND #11

### 2.5 Data collection and analysis

#### 2.5.1 Literature selection and data extraction

**Figure 1:**
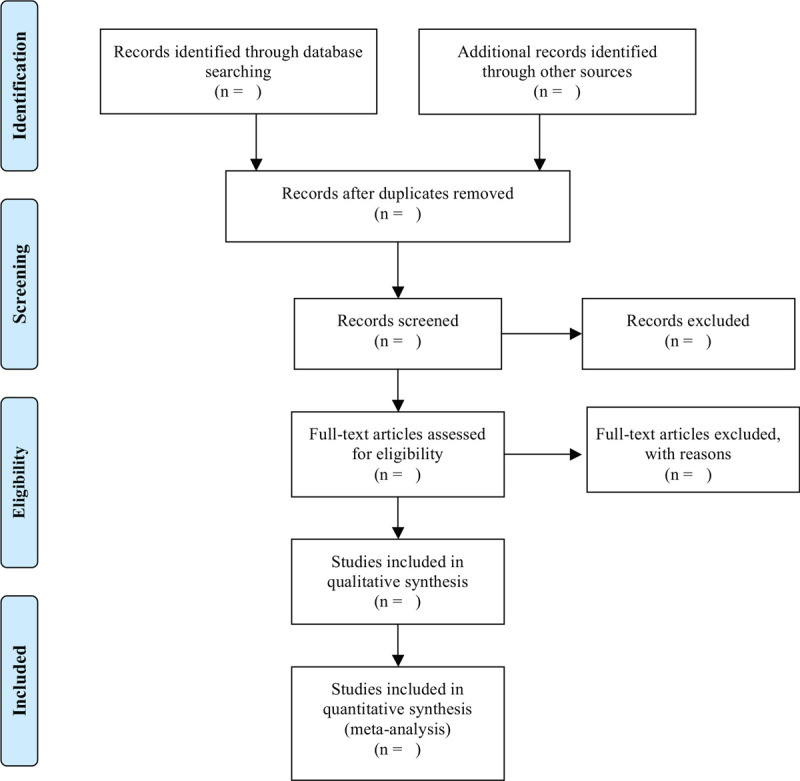
PRISMA flow diagram of the study selection process.

As shown in Fig. 1, 2 researchers (Peiyu He and Junyin Zhang) will screen the documents according to the inclusion and exclusion criteria: Import the retrieved documents into Endnote X9 software for review, then remove duplicate references; By preliminary screening abstract, we exclude documents, which obviously do not meet the inclusion criteria; Download and read the full paper for follow-up examination; After the final inclusion, we will use the pre-designed data extraction table for data extraction and cross-check the results; If there is any objection, the third researcher (Yingxiang Wang) will be asked to assist in the judgment. The main content of data extraction includes basic information of literature (title, journal, author, publication date), basic information of the research object (sample size, gender, average age, intervention, and course of treatment), and result data (numbers of response events, nonresponse events, dropouts, time points, mean, SD, follow-up time, and adverse events). If the required information is missing or incomplete, we will contact the relevant email address of the corresponding author or first author of the original document. If the relevant data cannot be obtained, the record is excluded. At the same time, the key factors of bias risk assessment are extracted.

#### 2.5.2 Risk of bias assessment

The method quality of systematic review reflects the risk of deviation or validity in its process and results. The quality of the method will be evaluated according to Cochrane Manual 5.2.0.^[18]^ Two well-trained researchers (Peiyu He and Junyin Zhang) independently assessed the risk of bias in the study. The evaluation content includes generation of random sequences, randomization concealment, the implementation of blinded subjects and researchers, the implementation of blind methods for outcome evaluators, the integrity of outcome data, selective reporting, and other biases. Each item should be judged as 3 “low risk” levels bias, “high-risk bias,” and “unclear” follow quality classification standards. For each item, if it is satisfactory, it means “low risk deviation,” if not, it means “high risk deviation.” When there is not enough information in the literature to make a clear judgment on the corresponding item, it means “unclear.” If there is any dispute, we will submit it to the corresponding author (Teng Peng) for arbitration.

#### 2.5.3 Data synthesis

Meta-analysis will be performed by RevMan 5.0 software (Version 5.3, Copenhagen: The Nordic Cochrane Center, 2014) provided by the Cochrane Collaboration. Although there is statistical homogeneity between each study (*I*^2^ <50%), the fixed effect model is used. When the heterogeneity is significant (*I*^2^ ≥50%) between the results of each study, the sublayer analysis is performed to find the source of heterogeneity. A fixed effect model is used for meta-analysis when there is sufficient similarity between the results of the subgroups (*I*^2^ <50%). However, a random effect model is used for meta-analysis if the heterogeneity between the results of the subgroups is significant (*I*^2^ ≥50%). Qualitative heterogeneity is used when heterogeneity is too large or the source of heterogeneity is unknown. Meta-regression analysis can be performed if there are many influencing factors and it is not appropriate to use the stratification method.

#### 2.5.4 Assessment of heterogeneity

Before the combination of effect size, the heterogeneity of the included literature is tested using Stata 13.0. When inter-study heterogeneity exists, the random effect model will be used. For comparison of each pair, heterogeneity is determined by heterogeneity test and expressed by *I*^2^ value. When *I*^2^ ≥50%, the heterogeneity is large. When 25%<*I*^2^<50%, we think it is moderate. When *I*^2^ <25%, the heterogeneity is considered small.

#### 2.5.5 Sensitivity analysis

Sensitivity analysis is based on sample size, missing data results, and methodological quality. Sensitivity analysis will be put into effect to examine the robustness of the pooled results in case of sufficient data by determining the effects of excluding studies with high risks of bias or with missing data, and outliers.

#### 2.5.6 Subgroup analysis

Subgroup analysis is to explore the source of heterogeneity. When more than 10 studies are included, subgroup analyses will be performed based on different participants, gender, duration of disease, interventions, and dose. We can better explore the source of heterogeneity as investigator by this method.

#### 2.5.7 Assessment of reporting bias

If 10 or more papers are conducted, a comparison-adjusted funnel plot is developed to using Stata to evaluate the presence of small sample effects or publication bias in the intervention. Descriptive analysis will be carried out by the method of the symmetry of funnel plot. If there is asymmetric or no inverted funnel in the plot, it is deemed that there may be publication bias. It is possibly connected with the difficulty in the publication of the literature with negative results and the low quality of the inclusion methods.

#### 2.5.8 Grading the quality of the evidence

To grade evidence quality and understand the actual situation of evidence rating thereby analyzing possible questions, the Grading of Recommendations Assessment, Development and Evaluation (GRADE) system will be used to evaluate the quality of evidence.^[19]^ On account of the risk of bias, imprecision, inconsistency, indirection, and publication bias, GRADE grades evidence quality into 4 levels: high, medium, low, and very low.

## 3 Discussion

Diabetes is a metabolic disease characterized by chronic hyperglycemia, which is induced by genetic or environmental factors, accompanied by insufficient or relatively insufficient insulin secretion, causing glucolipid metabolism disorders.^[20]^ Insulin, sulfonylureas, and biguanides are traditional hypoglycemic drugs, these drugs are effective in lowering the glucose level of blood, but they also have some side effects and body dependence can develop.^[9]^ More studies have shown that HYS not only can control blood glucose levels, but also can effectively alleviate the symptoms of patients with type 2 diabetes. However, there has been no systematic review or meta-analysis to evaluate its efficacy and safety. Therefore, it is necessary to provide convincing evidence for the superiority of HYS in hyperlipidemia through high-quality systematic review and meta-analysis. In addition, the study may have potential drawbacks. First, the Chinese-English research format is likely to increase the research bias. Second, age, gender, ethnicity, drug formulation, dose, and duration of treatment led to the risk of heterogeneity. Finally, this study may involve a small number of clinical trials, leading to a high risk of bias.

## Author contributions

Peiyu He and Junyin Zhang made the same contribution to the research and design, and wrote the original draft of the protocol. Peiyu He has developed a search strategy. Peiyu He, Junyin Zhang, and Yingxiang Wang will conduct literature retrieval and collation. Peiyu He, Junyin Zhang, and Teng Peng will evaluate the risk of bias in the literature. Data analysis and article writing will be done by Peiyu He, Tianyu Gao. Teng Peng, as the corresponding author, will be responsible for overseeing every process of the audit review to control the quality of the study. All the authors have approved the publication of the protocol.

**Data curation:** Teng Peng.

**Methodology:** Yingxiang Wang.

**Project administration:** Tianyu Gao.

**Writing – review & editing:** Peiyu He, Junyin Zhang.
